# Supplementary material for: Regional variation in COVID-19 positive hospitalisation across Scotland during the first wave of the pandemic and its relation to population density: A cross-sectional observation study
Source: PLoS One. 2021 Jul 9;16(7):e0253636. doi: 10.1371/journal.pone.0253636 (PMC8270435; doi:10.1371/journal.pone.0253636)
Supplement: Attachment — Submitted filename: PLOSreview_comments to authors.docx [file pone.0253636.s004.docx]

Limitations:

- The conclusion is inappropriate and even dangerous because so many variables were not included in the data analysis such as co-morbidity, PH prevention measures such as quarantine, wearing facial masks and social distancing, lock downs, and spread of COVID through the different health regions during the pandemic.
- Another major issue is that you do not include hospital distribution in these health regions. Hospital distribution and the number of hospital beds or ICU beds or “Covid ward” is a function of population density and therefore this should be checked for multi-collinearity with variance inflation factor (VIF). I would argue the same is true for co-morbidities, age, socio-economic status (not able to work from home) instead of just median age.
- I am also not sure if COVID patients were always hospitalized in their own health region. You mention that briefly but maybe you would be able to quantify that and also how that changed during the pandemic. We had many patients’ transfers in the height of the pandemic to neighboring regional hospitals.
- What about the impact of nursing home or long-term care outbreaks, clearly a population who is at highest risk for hospitalization (and death)?
- The hospitalization rate is inappropriate. You are looking at persons hospitalized and not hospital admissions, correct? Therefore, the percentage of person hospitalized instead of rate should be a better measure.
- I think also time is another important variable to be included. We experienced in Louisiana a first wave of almost close to capacity hospitalizations in New Orleans but then the pandemic moved quickly into more rural parts of the state where it most likely spread through households and worksites or houses of religious worship (based on outbreaks identified)

Minor limitations:

- Cross-sectional study OR is really a prevalence odds ratio (POR)
- Table 1: do not use the term “cases” here since this is confusing, you talk about COVID hospitalizations.
- Reference 14 should be removed. Southwest Louisiana was impacted by two major hurricanes in the midst of the pandemic and experienced a surge in COVID cases, no hospital data available because no hospitals were left. Because of the damage and impact of these 2 storms residents had to evacuate to their extended family or hotels and live in crowded homes so their exposure risk (no social distancing or wearing face masks) increased dramatically plus these evacuees were not inclined to get tested because they had other major issues to deal with..
